# Arylsulfatase A (ASA) in Parkinson’s Disease: From Pathogenesis to Biomarker Potential

**DOI:** 10.3390/brainsci10100713

**Published:** 2020-10-07

**Authors:** Efthalia Angelopoulou, Yam Nath Paudel, Chiara Villa, Christina Piperi

**Affiliations:** 1 Department of Biological Chemistry, Medical School, National and Kapodistrian University of Athens, 11527 Athens, Greece; angelthal@med.uoa.gr; 2 Neuropharmacology Research Laboratory, Jeffrey Cheah School of Medicine and Health Sciences, Monash University Malaysia, Bandar Sunway, Selangor 47500, Malaysia; yam.paudel@monash.edu; 3 School of Medicine and Surgery, University of Milano-Bicocca, 20900 Monza, Italy

**Keywords:** PD, Arylsulfatase A, lysosomes, GWAS, Gaucher’s disease, prognostic biomarker

## Abstract

Parkinson’s disease (PD), the second most common neurodegenerative disorder after Alzheimer’s disease, is a clinically heterogeneous disorder, with obscure etiology and no disease-modifying therapy to date. Currently, there is no available biomarker for PD endophenotypes or disease progression. Accumulating evidence suggests that mutations in genes related to lysosomal function or lysosomal storage disorders may affect the risk of PD development, such as *GBA1* gene mutations. In this context, recent studies have revealed the emerging role of arylsulfatase A (ASA), a lysosomal hydrolase encoded by the *ARSA* gene causing metachromatic leukodystrophy (MLD) in PD pathogenesis. In particular, altered ASA levels have been detected during disease progression, and reduced enzymatic activity of ASA has been associated with an atypical PD clinical phenotype, including early cognitive impairment and essential-like tremor. Clinical evidence further reveals that specific *ARSA* gene variants may act as genetic modifiers in PD. Recent in vitro and in vivo studies indicate that ASA may function as a molecular chaperone interacting with α-synuclein (SNCA) in the cytoplasm, preventing its aggregation, secretion and cell-to-cell propagation. In this review, we summarize the results of recent preclinical and clinical studies on the role of ASA in PD, aiming to shed more light on the potential implication of ASA in PD pathogenesis and highlight its biomarker potential.

## 1. Introduction

Parkinson’s disease (PD) is the most common neurodegenerative movement disorder, affecting approximately 1–2% of the population above the age of 60 years [1]. Idiopathic PD represents the most common cause of parkinsonism, which is a clinical syndrome encompassing a number of nosologic entities sharing mainly three cardinal motor features: bradykinesia, resting tremor and rigidity [2]. Less common parkinsonian disorders include other neurodegenerative diseases, such as multiple system atrophy (MSA) and progressive supranuclear palsy (PSP), drug-induced and vascular parkinsonism [2]. PD is a progressive disorder characterized by motor and non-motor symptoms, including cognitive impairment, depression and autonomic dysfunction [1]. Currently, there is no disease-modifying therapy against PD, and its treatment remains mainly symptomatic.

PD is not a single entity but rather a clinically heterogeneous disorder with diverse subtypes including tremor-dominant, postural instability-gait difficulty and akinetic-rigid forms of PD, and there is still no consensus for clinical PD sub-classification [3,4,5]. Most cases of PD are sporadic, while only 5-10% are caused by known inherited mutations in specific genes, including *SNCA,* leucine-rich repeat kinase 2 *(LRRK2), Parkin (PARK2)* and phosphatase and tensin homolog (PTEN)-induced putative kinase 1 *(PINK1)* [6]. Apart from *LRRK2* mutation carriers, who are mostly indistinguishable from idiopathic PD reflecting the majority of genetic PD cases, display atypical features [6]. In particular, PD patients carrying *SNCA* mutations have an earlier age at disease onset, a faster motor deterioration, earlier cognitive decline and prominent multimodal hallucinations (visual, olfactory, auditory) [7]. *Parkin* mutation carriers display an earlier onset age of the disease, more frequently associated with dystonia as an initial manifestation, with dementia being less common despite the long disease course [8]. Notably, the pathophysiological mechanisms underlying this clinical diversity are still unclear, and there is still no available biomarker that can effectively distinguish between PD endophenotypes or reflect disease progression [4,5].

The neuropathological hallmarks of PD include the degeneration of dopaminergic neurons in the substantia nigra pars compacta (SNpc) and the accumulation of Lewy bodies and Lewy neurites mainly consisting of α-synuclein [9,10]. Although the etiology of PD remains obscure, mitochondrial dysfunction, abnormal α-synuclein aggregation, excessive neuroinflammation, lysosomal impairment and dysregulation of lipid metabolism contribute to its pathogenesis. Of note, α-synuclein is degraded via both proteasomal and autophagic-lysosomal pathways, and it may itself impair lysosomal activity [11]. Furthermore, lysosomal dysfunction has been shown to contribute to cell-to-cell spreading of α-synuclein aggregates in the brain, a process highly implicated in PD progression [12].

The core of Lewy bodies contains large amount of lipids coated with high local concentrations of non-fibrillar α-synuclein [13]. α-synuclein is able to interact with fatty acids and phospholipids [14], and the imbalance of α-synuclein-lipid interaction has been proposed to affect its aggregation [15]. Genome wide association studies (GWAS) have revealed that several genetic loci associated with PD risk are implicated in lipid metabolism, including *GBA1*, diacylglycerol kinase (*DGKQ*) and the phospholipase *PLA2G6* genes [16].

Among the causative genes of PD, *SNCA*, *LRRK2*, *PARK2*, *PINK1* and *ATP13A2 (PARK9),* are highly implicated in lysosomal function. In particular, α-synuclein aggregates have been demonstrated to impair autophagic-lysosomal pathways, either via direct disruption of lysosomal components or indirectly by suppressing lysosomal trafficking [17]. LRRK2 protein can modulate lysosomal vesicular trafficking by phosphorylating Rab GTPases [17], while *PARK2* and *PINK1* mutations have been associated with endo-lysosomal defects [18]. Moreover, the *ATP13A2* gene encodes a lysosomal P-type ATPase that is highly implicated in cation homeostasis, while *ATP13A2* mutations have been associated with mitochondrial and lysosomal dysfunction [19].

GWAS have revealed at least 24 genetic loci that may alter PD risk, and many of them, such as *SLC17A5*, *ASAH1* and *CTSD* have been implicated in the autophagy-lysosomal pathways [20,21,22]. Notably, heterozygous mutations in the *GBA1* gene encoding glucocerebrosidase, whose homozygous mutations cause Gaucher’s disease, constitute the most common genetic risk factor for idiopathic PD [23,24]. The presence of *GBA1* mutations has been also shown to affect the clinical phenotype of PD with a more rapid disease progression and cognitive impairment [25]. In addition, mutations in the NPC intracellular cholesterol transporter 1 *(NPC1)* gene, which cause the lysosomal disorder Niemann-Pick type C, have been associated with PD [26]. A recent study demonstrated that the majority of patients carry at least one potentially damaging variant in lysosomal storage disorder-related genes, and approximately one fifth of them possess multiple alleles [22]. The *ATP13A2* gene mutations, involved in a rare form of juvenile-onset parkinsonism and dementia, have been associated with the lysosomal storage disorder neuronal ceroid lipofuscinosis [27]. Hence, lysosomal impairment is suggested to play a crucial role in PD development, either as a causative or a contributing factor, with gene mutations causing other lysosomal storage disorders possibly affecting PD susceptibility.

Lysosomal storage disorders belong to the Mendelian-inherited metabolic diseases that are characterized by defects in the activity of lysosomal enzymes and the abnormal accumulation of undegraded substrates in the lysosomes of various tissues, including the heart, skin and brain [22,28]. Neurological manifestations, such as mental retardation, epilepsy and parkinsonism have been described in more than two-thirds of lysosomal storage disorders [28], including gangliosidosis, Niemann-Pick disease and Fabry–Anderson disease [29]. Furthermore, higher levels of α-synuclein oligomers have been detected in the plasma of patients with lysosomal storage disorders including Gaucher’s disease, Niemann-Pick type C, Krabbe disease and Wolman disease compared to controls, while this difference is absent in patients with Gaucher’s disease after enzyme replacement therapy [30]. These findings further strengthen the hypothesis that impairment of lysosomal enzymes may play a significant role in the pathogenesis of PD.

Arylsulfatase A (ASA) is a lysosomal enzyme that mainly hydrolyzes sulfatide (sulfogalactosylceramide), a glycolipid of myelin into galactosylceramide [31,32]. ASA is encoded by the *ARSA* gene, which is located on the chromosome locus 22q13.33. Homozygosity for *ARSA* mutations leads to severe ASA deficiency (<10%) that causes metachromatic leukodystrophy (MLD), a lysosomal storage disease that is inherited in an autosomal recessive manner with a reported frequency of 1:40000 [32,33]. MLD is characterized by abnormal accumulation of sulfatide primarily in the central nervous system (CNS), resulting in demyelination accompanied by motor and cognitive impairment [34,35].

Apart from sulfatide, ASA can also hydrolyze the sulfated glycolipids seminolipid and lactosylceramide sulfate [36]. Seminolipid exists only in small amounts in rat and mouse brain, whereas lactosylceramide sulfate was not detected in mammalian brain [36]. In addition to glial cells, sulfatide was present in neurons of *ARSA*-deficient mice [36], and neuronal sulfatide accumulation was associated with degeneration of Purkinje cells, axonal degeneration and cortical hyperexcitability [37]. Neuronal sulfatide storage is most prominent in the nuclei of medulla oblongata, pons and midbrain of *ARSA*-deficient mice [38], implying the potential role of ASA in neuronal functions unrelated to myelination. Notably, reduced levels of sulfatides by approximately 30% have been observed in the superior frontal and cerebellar gray matter of incidental PD human cases (with no complains of neurological deficits, although PD-related lesions were found in the neuropathological postmortem study) [39], suggesting that dysregulation of sulfatide metabolism may be implicated in the pathogenesis of PD.

ASA deficiency may be observed in healthy individuals (pseudodeficiency) displaying about 10–20% of normal enzymatic activity [33,34]. Partial ASA deficiency may result from homozygosity for the pseudodeficient *ARSA* allele, heterozygosity for a disease-causing *ARSA* allele or compound heterozygosity pseudodeficiency/deficiency. It still remains unclear whether partial ASA deficiency is completely benign [40]. Moreover, complete or partial ASA deficiency has been associated with parkinsonism and other movement disorders, including chorea, athetosis, dystonia, as well as several neurological conditions [29,41,42]. These findings suggest that ASA may also play a role in PD pathology and has led to an increasing number of preclinical and clinical studies investigating this relationship.

Although the potential implication of lysosomal ceramide metabolism disorders in PD pathogenesis has been already discussed in the literature [33], there is no recent review focusing specifically on the role of ASA in PD. Herein, we summarize recent emerging clinical and preclinical evidence on the possible role of ASA in PD and its biomarker potential, aiming to shed more light on the underlying mechanisms and their clinical impact.

## 2. Clinical Evidence on the Emerging Role of ASA in PD

### 2.1. ASA Levels and Activity as a Potential PD Biomarker

Reduced GBA activity has been detected in the cerebrospinal fluid of PD patients in comparison to controls [43] and in the blood of PD patients with and without *GBA1* mutations [44]. The activity of β-galactosidase and β-hexosaminidase, two other lysosomal enzymes, has been detected elevated in the CSF or blood of PD patients [43,45,46]. Therefore, it has been hypothesized that other lysosomal enzymes may be differentially expressed or display altered activity in PD patients in comparison to controls, independently of the presence of *ARSA* gene mutations.

Indeed, enzymatic activity of ASA in blood leukocytes has been shown significantly lower in patients with movement disorders, including essential tremor or parkinsonism, as compared to healthy controls or neurological patients without any movement disorder [47]. In particular, patients with atypical clinical features displayed reduced enzymatic ASA activity, while patients with typical essential tremor or parkinsonism showed normal values of ASA activity [47]. Atypical manifestations included pyramidal signs, orthostatic and continuous tremor (postural and resting tremor with bilateral onset of the same large amplitude), focal dystonia and/or no treatment response to levodopa, primidone or phenobarbitone [47]. Conventional neuroimaging of these patients with CT brain scans did not reveal any significant structural abnormalities [47]. Notably, a positive family history for movement disorders was mainly reported in the subgroup of patients with atypical symptoms [47], highlighting the potential contribution of *ARSA* gene polymorphisms or other inheritable factors affecting ASA activity in this clinical variability. A retrospective pedigree analysis of three of the patients with parkinsonism, positive family history and partial ASA deficiency demonstrated that the affected relatives displayed also partial ASA deficiency [40]. These patients exhibited atypical clinical features, such as head tremor, early postural or kinetic tremor, orthostatic tremor, cognitive impairment, and mild to moderate levodopa response [40]. Therefore, ASA deficiency may be possibly associated with an atypical PD clinical phenotype, which may also reflect a different pathophysiological background.

Postural tremor was the initial symptom in almost all cases of the study described above, and the only manifestation in a young patient [40]. It has been suggested that essential tremor may represent a potential “risk factor” for PD development, although the underlying pathophysiological mechanism remains unclear [48]. Given the results of the study reported above, it can be speculated that ASA deficiency might partially underlie this observed clinical association in some cases.

Interestingly, early cognitive impairment has been associated with *GBA1*-related PD [23,24,49], postural and action tremor has been reported in asymptomatic carriers of *LRRK2* mutations [50], and PD patients with *SNCA* mutations display early dementia [51]. These clinical observations suggest that mixed tremor and early cognitive decline may characterize PD cases related to genes associated with lysosomal function.

A recent study has demonstrated that plasma ASA levels were lower in PD patients with dementia compared to controls, whereas PD patients without dementia displayed higher plasma levels [31]. Moreover, plasma ASA levels were positively correlated with the scores of global cognitive performance, total Mini-Mental State Examination (MMSE) score and each cognitive domain separately, except for visuospatial function [31]. In PD patients, the clinical Dementia Rating-Sum of Boxes (CDR-SOB) scores were also negatively correlated with plasma ASA levels [31]. The different ASA levels between PD patients and controls, as well as PD patients with and without dementia, further highlight the contribution of ASA to PD pathogenesis and clinical endophenotypes.

Notably, a very recent study has demonstrated an interesting relationship between ASA plasma levels and PD progression [52]. In particular, the early PD subgroup characterized by shorter disease duration and lower UPDRS motor scores had higher ASA plasma levels, as compared to the late PD subgroup and healthy controls, independent of age, gender and MMSE scores [52]. Plasma ASA levels were graphically represented as an inverted U-shape in regard to the duration of the disease, reaching a peak at approximately 2 years of disease duration [52]. In the early PD subgroup, plasma ASA levels were also positively correlated with UPDRS motor scores and striatal dopamine depletion as evaluated by DATscan [52]. Other demographic or clinical parameters, such as age at disease onset, education years or MMSE scores, were not associated with plasma ASA levels [52]. These findings partially agree with the results of the abovementioned study which showed that PD patients without dementia displayed increased ASA levels, compared to PD with dementia or healthy controls [31]. Plasma ASA levels may increase at the early stages of PD in relation to nigrostriatal degeneration possibly reflecting an initial compensatory mechanism in response to accumulation of aggregated α-synuclein in neurons [52]. In agreement with this evidence, the concentration of plasma lysosomal enzymes differed in AD patients in regard to disease progression [53]. Hence, the plasma concentration of ASA may not be constant but rather change dynamically throughout disease course, implying that it could be used as a PD biomarker of disease severity and/or duration. However, larger longitudinal studies with serial measurements of plasma ASA levels in PD patients are required to validate their actual alterations during disease progression [52].

Apart from the brain, ASA is also expressed in the spinal cord, blood leukocytes and other peripheral tissues, and can be secreted by the cells [54]. It has been demonstrated that some serum exosomes may also contain lysosomal enzymes [55]. Nevertheless, the exact source of plasma ASA in both healthy controls and PD patients, as well as the proportional contribution of each source to plasma ASA concentration, remains to be elucidated [31]. Furthermore, it is largely unknown whether plasma ASA levels or activity correlate with the respective ASA levels or activity in the brain of PD patients and healthy controls.

It has been proposed that partial ASA deficiency alone or in combination with other yet unknown endogenous or exogenous factors may lead to dysfunction of specific neuronal cell populations that are particularly susceptible to metabolic alterations [56]. This hypothesis might at least partially explain the diverse and atypical parkinsonian clinical phenotype of patients with ASA deficiency caused by impairment of specific functional neuronal networks. Reduced GBA enzymatic activity has been reported in the brain of PD patients carrying *GBA1* mutations [57]. Decreased activity of GBA has been found in the SN and frontal cortex of patients with PD and LBD compared to controls [58]. Different enzymatic activity of GBA has been demonstrated in the substantia nigra and cerebellum of the brain of PD patients [57], highlighting that GBA activity is rather brain region-specific. Hence, further post-mortem investigation of ASA activity in the different brain structures of PD patients may explain the potentially ASA-related atypical parkinsonian clinical features.

Importantly, it has been demonstrated that GBA activity in healthy subjects decreases in an age-dependent manner in the brain regions mostly affected by PD, and at the 7th to 8th decade of life, GBA activity in the SN and putamen is reduced to the same extent as in patients with sporadic PD [59]. Therefore, GBA activity seems to be age-dependent even in the brain, and this hypothesis should be also taken into account and investigated in future studies exploring the role of ASA in PD.

In summary, ASA deficiency may be at least partially responsible for some PD cases with atypical symptoms, and represent a potential biomarker for clinical PD endophenotypes, mainly including patients with early cognitive dysfunction and early postural or mixed tremor. In addition, ASA activity may also aid in the differential diagnosis between PD and essential tremor, especially in cases with atypical presentation. However, given the small sample size of the abovementioned studies, further larger validation is needed to test these hypotheses.

### 2.2. ASA Localization in the Brain of PD Patients

Deposition of α-synuclein has been demonstrated in brain cells of patients with Gaucher’s disease [60], and α-synuclein accumulation has been shown to be diffuse in the axons of neurons and glial cells of the cerebral white matter and brainstem of MLD patients [61]. α-synuclein-immunoreactivity colocalized with abnormal storage products including lipids in the brain of MLD patients, suggesting that impaired lipid metabolism may affect α-synuclein deposition in these cases [61]. Additionally, a granular pattern of α-synuclein appeared in the cytoplasm without fibrils in the aforementioned study [61]. In vivo evidence has indicated that α-synuclein can interact with polyunsaturated fatty acids resulting in the production of α-synuclein soluble oligomers, a process that precedes the formation of aggregates associated with neurodegeneration [62]. Hence, it seems that in MLD, α-synuclein accumulates in the cytosol without fibril formation, through potential binding to fatty acids.

α-synuclein was also found to accumulate in the neurons and glial cells of MLD cases, including astrocytes and microglia [61] while it can act directly on microglia, initiating a neuroinflammatory response and affecting neuronal survival [63]. In parallel, microglia are also involved in clearing of the extracellular α-synuclein aggregates by internalization and degradation, thus avoiding its accumulation [63]. Given the importance of neuroinflammation in PD pathogenesis, the role of ASA in neuron-to-microglia communication should be further explored.

Collectively, these findings suggest that α-synuclein accumulation may accompany MLD-related neuropathology, strengthening the hypothesis that ASA might be possibly implicated in PD-related α-synuclein deposition in the human brain.

In this context, post-mortem investigation of brain tissues derived from the anterior cingulate cortex of PD patients and age-matched healthy controls has indicated that ASA was present in neuronal and glial cells, as well as in cells of blood vessels in PD patients and controls [31]. Interestingly, ASA had a puncta (particle-like) appearance, and it was localized throughout the cytoplasm of neurons, surrounding or sometimes co-localized with Lewy bodies, particularly in cases with longer disease duration [31]. However, no significant differences were detected in either the neuronal intensity of ASA fluorescence signal or the number of ASA-stained neurons between PD patients and controls in this study [31]. Although interesting, these findings only demonstrate that ASA and Lewy bodies share a cytosolic localization, and therefore, more evidence is needed before suggesting that ASA may contribute to α-synuclein-containing Lewy bodies in PD.

### 2.3. Possible Association between ARSA Gene Variants and PD

The potential role of *ARSA* gene mutations in PD has been illustrated in a recent study that genetically analyzed a female patient of Japanese descent with adult-onset MLD and a positive family history of PD with mild cognitive dysfunction and probable essential tremor [31]. In particular, the MLD patient bears the compound heterozygous missense mutations, L300S (c.899T>C, rs199476389) and C174Y (c.521G>A, rs199476381) in the *ARSA* gene, while a heterozygous L300S mutation was found only in her family members with PD—her father and paternal uncle—but not in those without PD [31]. These findings suggest that the heterozygous L300S mutation in *ARSA* gene may be a risk factor for PD development, and that *ARSA*-related PD may be associated with early cognitive decline and essential tremor. However, the L300S *ARSA* variant was not detected in a case-control study in the Chinese population in either sporadic PD patients or healthy controls [64], implying that this variant might be pathogenic but rarely detected in PD. Moreover, the L300S and C174Y *ARSA* variants were not detected in more than 200,000 alleles in public databases, implying that these variants are very rare and indicating the need for larger multicenter studies in patients with familial and sporadic PD [31].

The abovementioned study has also genetically analyzed *ARSA* variants in a cohort of 92 patients with autosomal dominant familial PD and revealed a non-synonymous N352S variant (c.1055A>G, rs2071421) in both homozygous and heterozygous state in 4 and 11 patients, respectively, while the frequency of this variant was significantly higher in healthy controls within the Integrative Japanese Genome Variation Database [31]. Further analysis in another cohort of 92 patients with sporadic PD revealed that the frequency of the N352S variant was similar to that of the cohort of patients with autosomal dominant familial PD [31]. It has been demonstrated that the L300S *ARSA* mutation results in complete loss of ASA activity, while the N352S variant has no effect on ASA activity but rather leads to the loss of the N-glycosylation site of the enzyme [65]. Although the sample size of this study is relatively small, these results suggest that the N352S *ARSA* variant may represent a protective genetic factor against PD development.

On the contrary, a very large GWAS among patients with PD or other α-synucleinopathies (MSA, LBD and REM-sleep behavior disorder, RBD) and controls of European ancestry has found no significant associations between the N352S *ARSA* variant and these diseases [66]. In accordance, a large Japanese PD GWAS did not reveal any association between this genetic locus and PD [67]. The N352S *ARSA* variant is a frequent genetic polymorphism [66], and its potential association with PD would have been identified with relative certainty on a genome-wide level [66]. In accordance, a recent study of 407 sporadic PD patients and 471 healthy controls in the Chinese population did not demonstrate any association between the *N352S ARSA* variant and PD development [64]. However, given the fact that its frequency is highly variable among European and East Asian populations [66], a potential population-specific effect cannot be ruled out.

In conclusion, data supporting a possible genetic association between these *ARSA* variants and PD are still rather weak. Hence, additional larger case-control studies among both familial and sporadic PD cases and/or in other ethnic groups are required to determine whether N352S or additional *ARSA* variants are related to PD pathogenesis.

## 3. Preclinical Evidence on the Potential Role of ASA in PD Pathogenesis

Glucocerebrosidase deficiency has been shown to lead to lysosomal impairment, thus promoting the aggregation and propagation of α-synuclein [12]. Given the implication of ASA in lysosomal sphingolipid metabolism [31], it has been speculated that ASA deficiency and subsequent sulfatide accumulation may also contribute to α-synuclein aggregation and cell-to-cell propagation.

In this context, a very recent study has revealed that ASA might be able to inhibit α-synuclein aggregation and propagation by acting as a non-lysosome-related molecular chaperone that could interact with α-synuclein in the cytosol [31]. In particular, *ARSA* knockout in SH-SY5Y human neuroblastoma cell lines, which resulted in almost complete loss of ASA enzymatic activity and increased sulfatide levels, was associated with elevated intracellular and extracellular soluble and insoluble fractions of α-synuclein aggregates, in comparison to the wild-type cells [31]. Notably, cell-to-cell transfer of aggregated α-synuclein was also enhanced in *ARSA* knockout cells, implying that *ARSA* deficiency may promote α-synuclein aggregates generation, secretion and propagation in vitro [31]. ASA could also protect against α-synuclein fibrillation in a dose-dependent manner in vitro, further strengthening this hypothesis. The enhanced *ARSA* depletion-induced α-synuclein propagation was also confirmed in vivo, in *Caenorhabditis elegans* models [31]. These findings suggest that ASA deficiency may promote α-synuclein aggregation and cell-to-cell transmission, potentially contributing to PD pathogenesis and/or progression.

Further analyses aiming to identify the underlying molecular mechanisms demonstrated that the effects of ASA on α-synuclein aggregation and propagation were independent of lysosomes or its enzymatic activity, but they might be mediated by its function as a molecular chaperone [31]. More specifically, ASA was able to directly interact with α-synuclein in the cytosol of differentiated SH-SY5Y cells where it displayed no enzymatic activity as well as in the brain of A53T mutant α-synuclein transgenic and non-transgenic mice [31]. Interestingly, the protein encoded by the pathogenic variant L300S *ARSA* interacted weakly with α-synuclein, while the protein encoded by the protective variant N352S *ARSA* displayed a stronger binding affinity with α-synuclein in vitro, compared to the wild-type ASA [31]. Furthermore, the protein encoded by the wild-type or N352S *ARSA* variant significantly reduced α-synuclein aggregation and propagation, in comparison to the L300S *ARSA* variant in vitro [31]. In α-synuclein transgenic fly lines, motor impairment as assessed by the climbing activity was rescued by the expression of the wild-type or the protective N352S *ARSA* variants, while L300S *ARSA* expression was not able to reverse the motor deficits of these insects [31]. It was also indicated that L300S *ARSA* expression was associated with increased mRNA but reduced protein levels, implying a potential decreased translation rate [31].

Collectively, these results suggest that the wild-type and N352S *ARSA* variant may protect against α-synuclein aggregation and spreading by acting as a molecular chaperone of α-synuclein, whereas the L300S *ARSA* variant may exert opposing effects, thus enhancing α-synuclein aggregation and transmission. These mechanisms may underlie the protective and pathogenic role of N352S and L300S *ARSA* variants in the risk of PD development respectively, as indicated by the clinical evidence described above.

## 4. Discussion and Future Perspectives

**Figure 1:**
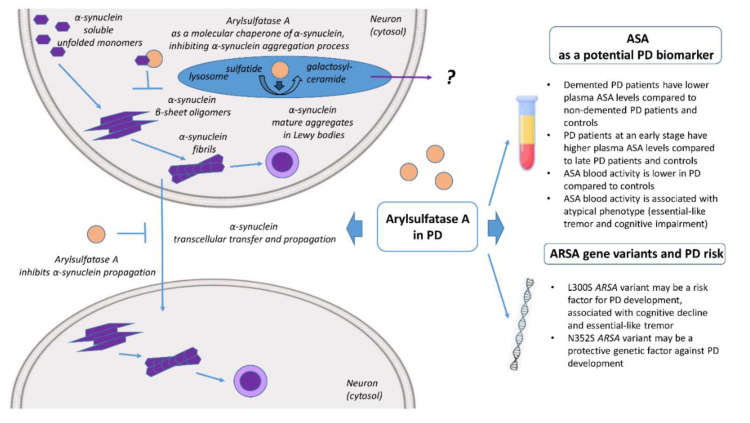
Implication of arylsulfatase A (ASA) in Parkinson’s disease (PD). In vitro and in vivo evidence has demonstrated that ASA may act as a molecular chaperone interacting with α-synuclein (SNCA) in the cytoplasm, thereby preventing its aggregation, secretion, and cell-to-cell propagation. Clinical evidence supports the role of ASA blood levels and activity as a potential biomarker for PD pathology, progression and PD phenotype. ARSA gene variants have been also shown to act as genetic modifiers of PD development.

In most cases, PD is considered a multifactorial disorder, since a complex interplay between several genetic and environmental factors may contribute to its development. Apart from the possibility of yet unknown genetic risk loci, epigenetic modifications may also account for familial forms of PD [22]. Potential interactions among rare or common alleles at various genetic loci may also be responsible for PD development [22], affecting the rate of disease progression or the clinical phenotype. Therefore, it is possible that specific *ARSA* gene variants may act in a “multi-hit” manner in combination with other yet unknown genetic or environmental factors, resulting in lysosomal impairment, α-synuclein aggregation and subsequent increased risk of PD (Figure 1).

Currently, there is no available biomarker for determining PD progression or distinguishing between different clinical endophenotypes. In this context, ASA levels and/or its enzymatic activity represent a promising candidate towards this direction (Figure 1). However, larger studies, investigating a combination of potential clinical, neuroimaging or other biochemical biomarkers may prove as a more appropriate approach [68].

In regard to the molecular mechanisms underlying the effects of ASA in PD, there is limited information up to date. ASA has been shown to act as a molecular chaperone of α-synuclein, thus inhibiting its aggregation. Of note, neuronal loss does not always correlate with sulfatide storage, as for instance Purkinje cells in the cerebellum degenerate in old ASA-deficient mice without any evidence for lipid accumulation [69]. However, this may rather not be the single underlying mechanism, given the multifaceted role of lipid metabolism in PD pathogenesis and the interaction of α-synuclein with fatty acids [15]. Although sulfatide is primarily found in oligodendrocytes, low amounts have been also detected in astrocytes and neurons [70]. It has been proposed that ApoE-containing lipoproteins secreted by astrocytes might take up sulfatide and get endocytosed by neurons via LDL receptor or LDL receptor-related proteins via an unknown mechanism [70]. This hypothesis could be further explored in the case of ASA deficiency also in PD. Notably, the amphipathic N-terminal domain of α-synuclein displays lipid binding properties and the missense mutations A53T and A30P of α-synuclein reside in the N-terminal domain [71]. Hence, the potential effects of ASA on mutated-α-synuclein-lipid interaction would further aid in the elucidation of its role in α-synuclein aggregation in PD.

Ambroxol, a pharmacological chaperone that binds to glucocerebrosidase, has been associated with motor improvement in animal models of PD [33,72]. A recent clinical trial has demonstrated that oral administration of Ambroxol was safe, well-tolerated and led to motor improvement in PD patients carrying or not *GBA1* mutations [72], paving the way for development of future therapeutic strategies based on lysosomic enzymes against PD. Another rational therapeutic approach is enzyme-replacement therapy with recombinant ASA, although crossing of blood–brain barrier represents a significant obstacle that would need to be overcome. In this context, a clinical trial has demonstrated that intrathecal delivery of recombinant human arylsulfatase A in children with MLD is well-tolerated [73]. Gene therapy via adeno-associated viral vectors for delivery of engineered DNA into cells is another promising treatment strategy that is currently under investigation for *GBA1*-related PD [74]. Finally, small molecules acting as enzyme enhancers, capable to cross the blood-brain barrier may also prove effective. Other small molecules targeting specific downstream pathways implicated in ASA function represent another candidate therapeutic target [75]. The appropriate choice of patients that are more likely to respond to a specific therapy is of paramount importance, since both carriers of *ARSA* variants as well as patients with sporadic idiopathic PD may benefit from ASA-related treatment approaches.

## 5. Conclusions

Emerging preclinical and clinical evidence supports the role of ASA in PD pathogenesis (Figure 1). ASA has been associated with an atypical PD clinical phenotype, including early cognitive impairment and essential tremor, as well as disease progression. Specific *ARSA* variants may act as genetic modifiers in PD, whereas there is also evidence that does not confirm this relationship. In vitro and in vivo evidence has also revealed that ASA may act as a molecular chaperone interacting with α-synuclein in the cytoplasm, thus preventing its aggregation, secretion and cell-to-cell propagation. Further larger, longitudinal, case-control studies in different ethnic populations are required for the clarification of the role of ASA in PD, given the advances on lysosome-targeted therapeutic strategies.

## Abbreviations

ASA: Arylsulfatase A; PD, Parkinson’s disease; SNCA, α-synuclein; SNpc, Substantia nigra pars compacta; LRRK2, *Leucine-rich repeat kinase 2; GWAS,* Genome wide association studies; MLD, Metachromatic leukodystrophy; CNS, Central nervous system; MMSE, Mini-Mental State Examination; CDR-SOB, Clinical Dementia Rating-Sum of Boxes; MSA, Multiple system atrophy; RBD, REM-sleep behavior disorder; LBD, Lewy body dementia.
